# Dynamic Observation of Interfacial IMC Evolution and Fracture Mechanism of Sn2.5Ag0.7Cu0.1RE/Cu Lead-Free Solder Joints during Isothermal Aging

**DOI:** 10.3390/ma13040831

**Published:** 2020-02-12

**Authors:** Di Zhao, Keke Zhang, Ning Ma, Shijie Li, Chenxiang Yin, Fupeng Huo

**Affiliations:** 1 School of Materials Science and Engineering, Henan University of Science and Technology, Luoyang 471023, China; 19B909143@stu.hit.edu.cn (D.Z.); lsxzmn@163.com (N.M.); lsjlishijieli@163.com (S.L.); yinyuxin416@foxmail.com (C.Y.); 2 Henan Province Key Laboratory of Nonferrous Metal Material Science and Processing Technology, Luoyang 471023, China; 3 State Key Laboratory of Advanced Welding and Joining, Harbin Institute of Technology, Harbin 150001, China; 4 Joining and Welding Research Institute, Osaka University, 11-1 Mihogaoka, Ibaraki, Osaka 567-0047, Japan; huofp@jwri.osaka-u.ac.jp; 5 Graduate School of Engineering, Osaka University, 2-1 Yamadaoka, Suita, Osaka 565-0871, Japan

**Keywords:** Sn2.5Ag0.7Cu0.1RE/Cu soldering, dynamic observation, isothermal aging, intermetallic compound, growth kinetics, fracture mechanism

## Abstract

Dynamic observation of the microstructure evolution of Sn2.5Ag0.7Cu0.1RE/Cu solder joints and the relationship between the interfacial intermetallic compound (IMC) and the mechanical properties of the solder joints were investigated during isothermal aging. The results showed that the original single scallop-type Cu_6_Sn_5_ IMC gradually evolved into a planar double-layer IMC consisting of Cu_6_Sn_5_ and Cu_3_Sn IMCs with isothermal aging. In particular, the Cu_3_Sn IMC grew towards the Cu substrate and the solder seam sides; growth toward the Cu substrate side was dominant during the isothermal aging process. The growth of Cu_3_Sn IMC depended on the accumulated time at a certain temperature, where the growth rate of Cu_3_Sn was higher than that of Cu_6_Sn_5_. Additionally, the growth of the interfacial IMC was mainly controlled by bulk diffusion mechanism, where the activation energies of Cu_6_Sn_5_ and Cu_3_Sn were 74.7 and 86.6 kJ/mol, respectively. The growth rate of Cu_3_Sn was slightly faster than that of Cu_6_Sn_5_ during isothermal aging. With increasing isothermal aging time, the shear strength of the solder joints decreased and showed a linear relationship with the thickness of Cu_3_Sn. The fracture mechanism of the solder joints changed from ductile fracture to brittle fracture, and the fracture pathway transferred from the solder seam to the interfacial IMC layer.

## 1. Introduction

In an electronic packaging system, solder joints provide an electrical connection and mechanical support to the electronic components. The need for function integration, high power, and density of the electronic products has been a driving force for highly reliable inter-connection solder joints [1,2]. Because of the inherent toxicity of lead, the use of SnPb solder has been restricted. Various types of environmental-friendly Sn-based lead-free alloys have been developed [3,4,5,6,7]. SnAgCu system lead-free solder alloys have been regarded as one of the promising candidates for SnPb solder alloys because of their good mechanical properties [8]. However, compared to the traditionally SnPd solders, the wettability SnAgCu lead-free solders are relatively poor. Additionally, the high Ag content of SnAgCu lead-free solders will lead to the formation of the brittle Ag_3_Sn phase. Apart from issues associated with properties and reliability, the cost is another issue to be considered. Rare earth (REs) also has a high chemical activity, which can effectively decrease the surface energy of molten alloys and thus improve the wettability of alloys. Xue and his team added Ce, Pr, and Nd in the SnAgCu solder alloys [9,10,11,12,13]. They found that the appropriate addition of Pr and Nd enhanced the shear bond strength of solder joints and refined the morphology of the interface layer. Yu et al. [14] found that with the addition of trace Ce and La elements, the β-Sn grains and eutectic colony in Sn3.5Ag0.7Cu alloy are refined. All these results indicated that adding trace rare earth elements was an efficient way to develop new solders. Our team has attempted to lower the Ag content by adding RE elements, obtaining quite promising results with the solder alloy of Sn2.5Ag0.7Cu0.1RE [15,16]. It is well known that the solder joints are frequently exposed to various harsh environments, such as temperature, humidity, shock, and vibration [17,18,19]. It was reported that approximately half the failure of the solder joints was caused by isothermal aging [20]. One of the most important reliability issues is the microstructure evolution of the solder joints, and understanding the growth behavior and kinetics of the interfacial intermetallic compound (IMC) is regarded as essential. In the literature, investigating the reliability of isothermal aging of Sn-based lead-free solder joints has become an area of interest. Many scholars have studied the isothermal aging characteristics of SnAgCu/Cu [21,22,23,24,25], In-48Sn/Cu [26,27], and Sn-Bi/Cu [28,29] solder joints. They have focused on the growth kinetics of the interfacial IMC and the mechanical properties of solder joints during isothermal aging. Xu et al. [21] investigated the influence of isothermal time and temperature on the interfacial IMC layer growth of Sn-3.5Ag-0.5Cu/Cu solder joints. Their studies showed that the interfacial IMC layer not only became thicker but also transformed from a scallop-like shape to planar shape with increasing isothermal aging time. Zhang et al. [22] reported the growth kinetics of the interfacial IMC layer of Sn-3.8Ag-0.7Cu/Cu solder joints and found that the IMC growth was controlled by the diffusion mechanism. Besides, they obtained the activation energy of the interfacial IMC. Hu et al. [23,24] further studied the relationships between the interfacial IMC and the fracture behavior of the Sn-3.0Ag-0.5Cu/Cu solder joints during isothermal aging. Nishikawa and Iwata [25] compared the reflow soldering and laser soldering and found that the growth of the interfacial IMC layer was slower by reflow soldering at the isothermal aging temperature 423 K. However, because of the slow reaction rate of the interfacial movement during isothermal aging, it is difficult to precisely measure the interfacial movement. To date, most methods used to investigate the growth kinetics and the interfacial movement during isothermal aging have used qualitative and statistical analysis [21,22,23,24,25], which cannot accurately reveal the constituents and growth of the interfacial IMC. Additionally, the relationship between the size and morphology of the interfacial IMC and the fracture mechanism of the solder joints during isothermal aging is not well understood.

Therefore, in this work, we conduct a dynamic observation of the interfacial IMC evaluation and the relationship between the growth behavior of interfacial IMC and the mechanical properties of the solder joints. There is important theoretical and practical value in predicting the reliability of solder joints.

## 2. Materials and Methods

### 2.1. Materials and Soldering

A Sn2.5Ag0.7Cu0.1RE solder alloy; pure metals of Sn, Ag, and Cu (purity 99.9%); and a RE mixture (with approximately 40% La and 60% Ce) were used as the raw materials. The solder alloy and Cu substrate were prepared according to the procedures described in reference [30]. After that, the soldering specimens were placed into the electric chamber furnace at 270 °C for 4 min.

### 2.2. Isothermal Aging

To illustrate the interfacial movement quantitatively, marks were carved on the Cu substrate and used as reference points. The indentation was carried out by the MHV-2000 Micro-Vickers (Laizhou Huayin Testing Instrument Co., Ltd., Laizhou, China). After the indentation test, a rectangular pyramid mark could be found on the Cu substrate. The marks made by micro-Vickers are assumed to be fixed during isothermal aging. The specimens were aged at 100, 125, 150, and 175 °C for 72, 192, 288, and 360 h and the morphology of the solder joints was observed for each aging time. Additionally, to reduce the oxidation of the surface, the specimens were placed in a vacuum electric chamber furnace with the argon gas atmosphere (purity > 99.99%).

### 2.3. Characterization Methods

The microstructure of the solder joints was obtained by a scanning electron microscope (SEM, JMS-5610LV, Tokyo, Japan), and the chemical composition was determined by energy dispersive spectroscopy (EDS, Inca X-sight, Oxford Instruments, Oxford, England), X-ray diffraction (XRD, Bruker D8-Advance, Billerica, MA, USA) patterns of the soldering samples were recorded on a Bruker D8 Advance X-ray diffractometer in 2θ ranging from 25° to 80°. The shear strength tests were carried out in a UTM2503 universal testing machine (Shenzhen SUNS technology stock Co., Ltd., Shenzhen, China) at room temperature with a rate of 1 mm/min.

**Figure 1:**
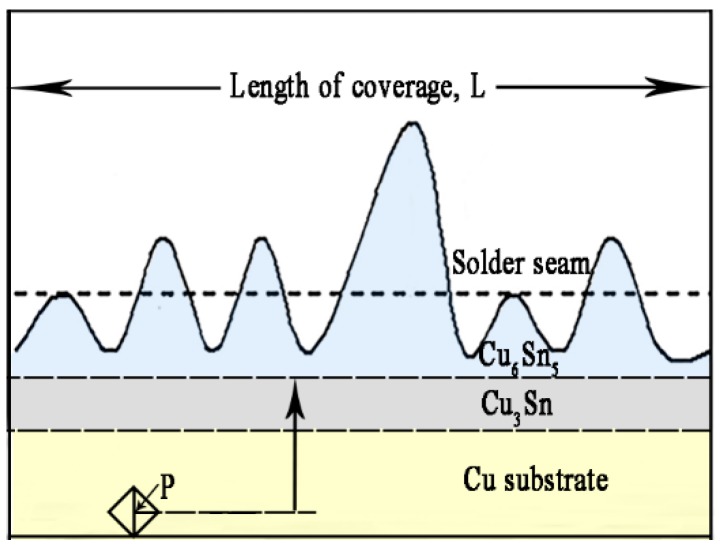
Schematic diagram of interfacial intermetallic compound.

To describe the growth behavior of the interfacial IMC layer and interfacial movement, the distances between the mark (“P”) and the interface of Cu/Cu_3_Sn, Cu_3_Sn/Cu_6_Sn_5_, and Cu_6_Sn_5_/solder were measured with increasing isothermal aging time, as illustrated in Figure 1. The distance between the interface and the marked point (“P”) was measured by the Image–Pro Plus 6.0 software (Media Cybernetics, Rockville, MD, USA). The average thickness of the interfacial IMC (*d*) was calculated by the Equation (1) [31]:

*d* = *A/L*, (1)

where *A* is the area of interfacial IMC layers and *L* represents the length of the coverage.

## 3. Results and Discussion

### 3.1. Interfacial IMC Evolution of the Sn2.5Ag0.7Cu0.1RE/Cu Solder Joints during Isothermal Aging

**Figure 2:**
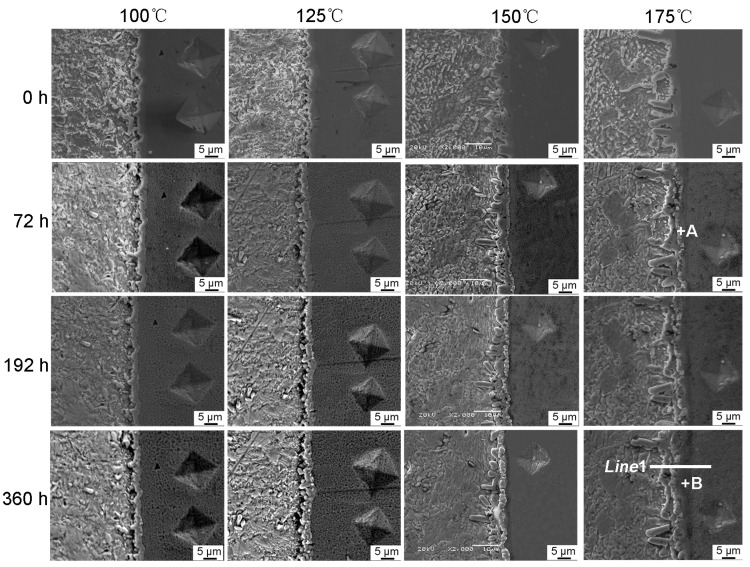
Cross-section micrographs of the interfacial IMC aged for different time and temperature.

**Figure 3:**
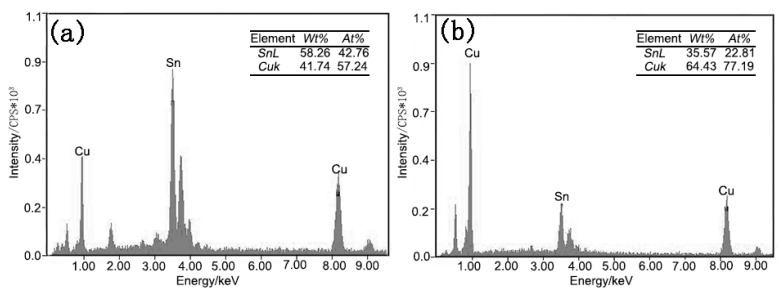
EDS analysis result of the point “A” and “B” in Figure 2. (**a**) point “A”; (**b**) point “B”.

**Figure 4:**
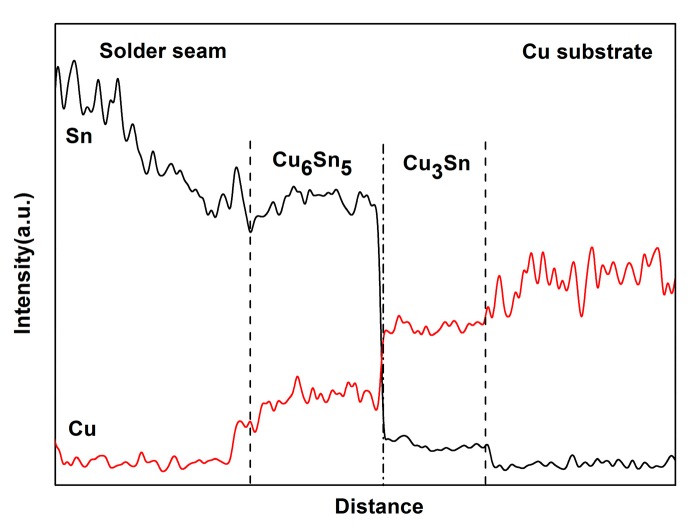
Elemental counts of the line “1” in Figure 2.

Figure 2 shows the dynamic observation interfacial microstructure evolution during isothermal aging. The solder joints consisted of a soldering seam, interfacial IMC, and Cu substrate. The soldered seam included the primary β-Sn phase and eutectic phases which were presented at the boundary of the primary β-Sn region. The eutectic phases included the fine acicular β-Sn+Ag_3_Sn, granular β-Sn+Cu_6_Sn_5_ binary eutectics, and the β-Sn+Ag_3_Sn+Cu_6_Sn_5_ ternary eutectic [32,33]. A continuous Cu_6_Sn_5_ interfacial IMC layer with a scallop-like morphology was formed between the Cu substrate and solder seam after soldering. In theory, the Cu_3_Sn IMC should exist between Cu and Cu_6_Sn_5_; however, Cu_3_Sn is often too thin after soldering to be detected by SEM [34]. With increasing isothermal aging time and temperature, the thickness of interfacial IMC layers clearly increased (Figure 2), and the morphology gradually evolved from scallop-type to planar because of the growth and the combination of adjacent scallop-like Cu_6_Sn_5_. In addition, the composition of the interfacial IMC also changed. After isothermal aging for 72 h at 175 °C, the scallop-like Cu_6_Sn_5_ covered a newly generated layer (marked “A”), which was determined to be the Cu_6_Sn_5_ phase by EDS analysis (Figure 3a). Additionally, a distinct dark grey region below the Cu_6_Sn_5_ layer appeared (marked “B”), which was confirmed to be the Cu_3_Sn phase by EDS analysis (Figure 3b). Figure 4 shows the distribution of Cu and Sn atoms of “line 1” in Figure 2 after isothermal aging at 175 °C for 360 h. It was obvious that the interfacial IMC layer consisted of two IMCs layers which were Cu_6_Sn_5_ and Cu_3_Sn IMC by the above analysis. This indicated that the double-layer IMC comprising Cu_6_Sn_5_ and Cu_3_Sn IMC gradually evolved from the original single Cu_6_Sn_5_ IMC. As can be seen from Figure 2, the growth rate of interfacial Cu_3_Sn IMC is not obvious as the isothermal aging temperature is less than 150 °C, while the Cu_3_Sn IMC is observed with an isothermal aging temperature greater than or equal to 150 °C.

**Figure 5:**
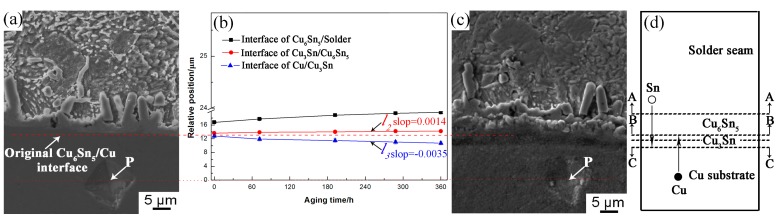
Interface position as a function of aging time and schematic illustration of the interfacial movement during aging: (**a**) after soldering, (**b**) interface position as a function of aging time, (**c**) isothermal aging 360 h, and (**d**) the schematic illustration of the interfacial movement.

**Figure 6:**
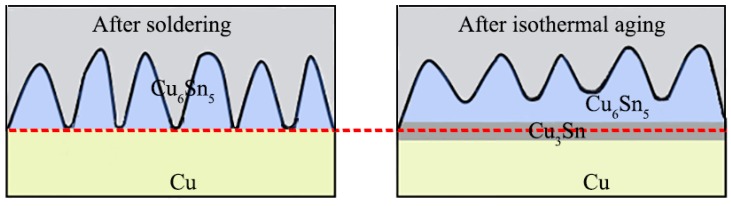
Schematic illustration of the interfacial IMC during isothermal aging.

To further analyze the growth behavior of interfacial Cu_6_Sn_5_ and Cu_3_Sn IMCs, we investigated the interface migrations during isothermal aging at 175 °C. Based on the above analysis, the Cu_3_Sn IMC was not detected after soldering. Thus, it was assumed that there were only solder seam/Cu_6_Sn_5_ and Cu_6_Sn_5_/Cu interfaces after soldering. To obtain the interfacial movement, the distances between the point “P” and the Cu/Cu_3_Sn, Cu_3_Sn/Cu_6_Sn_5,_ and Cu_6_Sn_5_/solder interface were measured with the increase of isothermal aging time, as shown in Figure 5b. Compared with the original Cu_6_Sn_5_/Cu interface in Figure 5a, when the isothermal aging time increased to 360 h (Figure 5c), the relative distance from the Cu/Cu_3_Sn, Cu_3_Sn/Cu_6_Sn_5_, and Cu_6_Sn_5_/solder interfaces to the point “P” tended to decrease, slightly increase, and substantially increase, respectively. This indicated that with increasing isothermal aging time, the thickness of Cu_6_Sn_5_ increased, the Cu_3_Sn IMC formed at the original Cu_6_Sn_5_/Cu interface, and the Cu_3_Sn IMC grew towards both the Cu substrate and Cu_6_Sn_5_ sides. During isothermal aging, the original Cu_6_Sn_5_/Cu interface disappeared and gradually formed a Cu_6_Sn_5_/Cu_3_Sn and Cu/Cu_3_Sn interface. This may be ascribed to the growth of interfacial IMC being dominated by the inter-diffusion of the Sn and Cu atoms during the isothermal aging process. Cu atoms from the Cu substrate diffused to the Cu_3_Sn/Cu_6_Sn_5_ interface, and the following reaction occurred: Cu_6_Sn_5_ + 15Cu = 5Cu_3_Sn (reaction 1) [35]. Additionally, some Sn atoms may diffuse from the solder seam to the Cu_3_Sn/Cu interface, and the reaction of 3Cu + Sn = Cu_3_Sn (reaction 2) occurred [35]. Therefore, the Cu_3_Sn layer grew to both sides, causing the Cu/Cu_3_Sn interface to move to the Cu substrate side and the Cu_6_Sn_5_/Cu_3_Sn interface to move to the Cu_6_Sn_5_ side during isothermal aging. Paul et al. [36] used ThO_2_ particles as markers at the Cu/Cu_6_Sn_5_ diffusion couple interface. After isothermal aging at 215 °C for 225 h, they found that the ThO_2_ particles were located in the inner Cu_3_Sn layer, indicating that Cu_3_Sn grew to both the Cu substrate and Cu_6_Sn_5_ side. Our observations here are consistent with their results. In addition, it was found that the linear slope of the Cu_3_Sn/Cu interface (line *l_3_* (Figure 5b)) was higher than that of the Cu_6_Sn_5_/Cu_3_Sn interface (line *l_2_*). This indicated that the growth rate of Cu_3_Sn on the Cu substrate side was higher than that on the side of Cu_6_Sn_5_ during isothermal aging. It was reported that the diffusion rate of Sn atoms from the solder seam to the Cu substrate was faster than that of the Cu atoms from the Cu substrate to the Cu_6_Sn_5_/Cu_3_Sn interface at the higher temperature (≥170 °C) in the Sn-Cu couple experiment [37]. Therefore, the growth rate of Cu_3_Sn on the Cu substrate side was higher than that on the side of Cu_6_Sn_5_ during isothermal aging. Figure 5d shows a schematic illustration of the interfacial movement of Sn2.5Ag0.7Cu0.1RE/Cu solder joints during isothermal aging. The arrow markers A-A, B-B, and C-C represent the movement direction of the solder seam/Cu_6_Sn_5_, Cu_6_Sn_5_/Cu_3_Sn, and Cu_3_Sn/Cu interfaces, respectively. We can conclude that the interface IMC becomes a double-layer consisting of Cu_3_Sn and Cu_6_Sn_5_ IMC, and the Cu_3_Sn IMC grows towards both the solder seam and Cu substrate simultaneously during the isothermal aging as illustrated in Figure 6.

### 3.2. Interfacial IMC Growth Kinetics of the Sn2.5Ag0.7Cu0.1RE/Cu Solder Joints during Isothermal Aging

**Figure 7:**
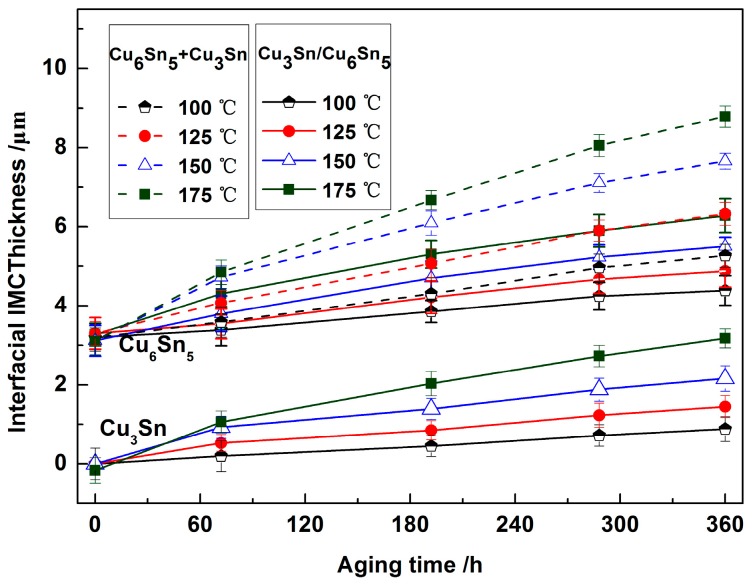
The thickness of interfacial IMC for different aging time and temperature.

Figure 7 displays the thickness of the interfacial IMC layer isothermal aged for different times and temperatures. The interfacial IMC evolved from a single Cu_6_Sn_5_ layer after soldering to a Cu_6_Sn_5_ and Cu_3_Sn IMC double layers during the isothermal aging process. At the isothermal aging temperatures of 100, 125, 150, and 175 °C, the thickness of Cu_6_Sn_5_ and Cu_3_Sn IMC increased with prolonged aging time. In addition, with increasing isothermal aging temperature, the thickness of Cu_6_Sn_5_ and Cu_3_Sn IMC also increased.

To further study the growth kinetics of Cu_6_Sn_5_ and Cu_3_Sn IMC, we investigated the growth behavior of Cu_6_Sn_5_ and Cu_3_Sn IMC during isothermal aging at 175 °C. During isothermal aging, the growth of the interfacial IMC layer had a diffusion-controlled mechanism, and the growth kinetics parameter was calculated by measuring the interfacial thickness as a function of the isothermal aging time. The relationship between the interfacial IMC layer thickness and the aging time can be expressed as follows [38]:

*d_x_* = *Kt^n^* + *d*_0_. (2)

In Equation (2), *d_x_* presented the thickness of the interface IMC layer at a time (*t*), *d*_0_ is the initial thickness of the interfacial IMC layer after soldering, *K* is the growth constant (mm^2^/s) that is related to the diffusion coefficient of the atoms, and n is the time exponent. The value of n can be determined by a multivariable linear regression analysis of Equation (2) when placed in the following format:

ln(*d_x_ − d*_0_) = ln*K* + *n*ln*t*. (3)

**Figure 8:**
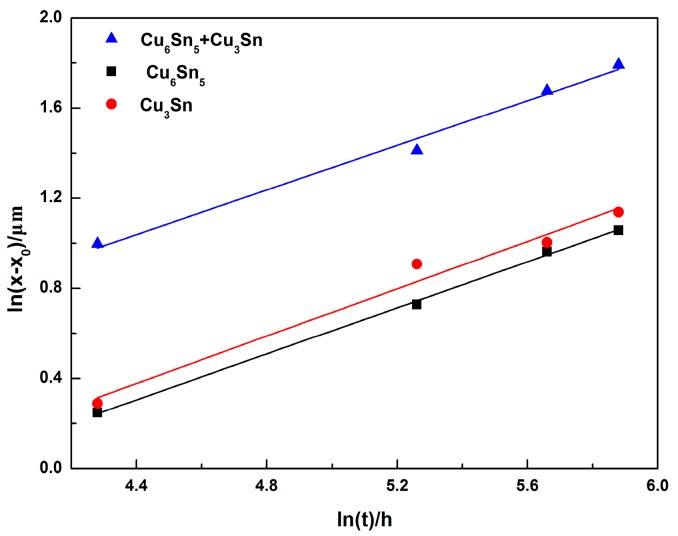
Plot of ln(*d_x_ − d*_0_) versus ln(*t*).

The time exponent n was obtained from the slope of the *ln*(*d_x_ − d*_0_) versus ln*t* plot in Figure 8. In this study, the values of the time exponent n of Cu_6_Sn_5_ + Cu_3_Sn, Cu_6_Sn_5_, and Cu_3_Sn were 0.49, 0.53, and 0.51, respectively, which are close to 0.5. It has been reported that if the value of the time exponent is 0.33, the growth of the interfacial IMC had a grain-boundary diffusion mechanism, whereas if the time exponent is 0.5, the growth of the interfacial IMC had a bulk diffusion mechanism [39]. This indicated that the growth of Cu_6_Sn_5_ and Cu_3_Sn IMC layer during the isothermal aging process was controlled by the bulk diffusion mechanism. Then, Equation (3) can be expressed as follows:

*d_x_* = *Kt*^1/2^ + *d*_0_. (4)

**Figure 9:**
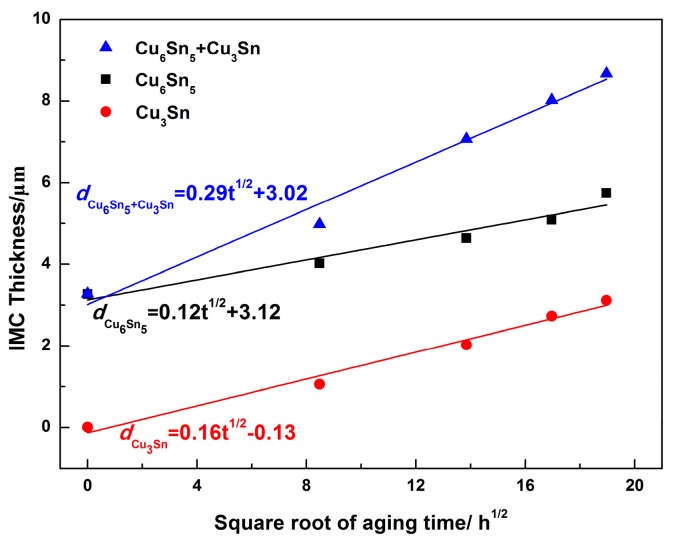
Linear fit of interfacial IMC thickness versus square root of aging time.

From Equation (4), the thickness of the interfacial IMC was plotted against the root of the isothermal aging time (*t*). Figure 9 shows the thickness of (Cu_6_Sn_5_ + Cu_3_Sn), Cu_6_Sn_5_, and Cu_3_Sn against the square root of the isothermal aging time. The thickness of the interfacial IMC showed a linear relationship. The value of the growth constant (*K*) was obtained by the slope of the linear regression. The growth constants of the interface (Cu_6_Sn_5_ + Cu_3_Sn), Cu_6_Sn_5_, and Cu_3_Sn were *K_Cu6Sn5+Cu3Sn_* = 2.34 × 10^−17^ m^2^/s, *K_Cu6Sn5_* = 6.25 × 10^−18^ m^2^/s, and *K_Cu3Sn_* = 7.11 × 10^−18^ m^2^/s, respectively. The growth constant of Cu_3_Sn was slightly higher than that of Cu_6_Sn_5_, which indicated that the growth rate of Cu_3_Sn was higher than that of Cu_6_Sn_5_ with aging time. This result was consistent with the dynamic observation of interfacial IMC growth.

**Figure 10:**
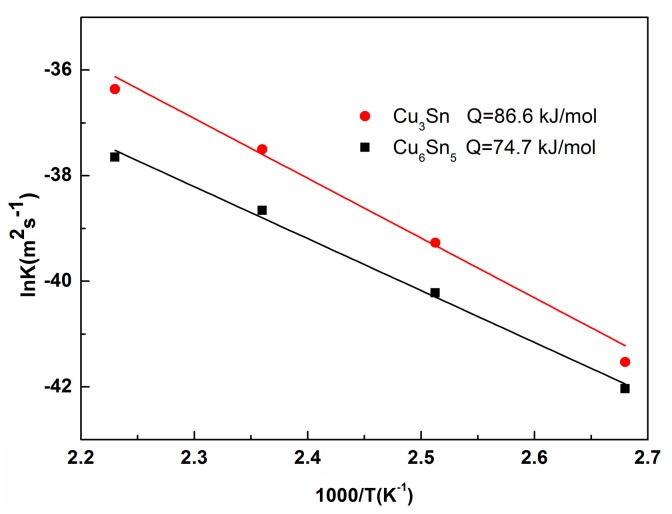
Arrhenius plot of the IMC layer growth during isothermal aging.

During the isothermal aging process, the growth of interfacial Cu_6_Sn_5_ and Cu_3_Sn IMC was controlled by the interdiffusion of Cu and Sn atoms. The activation energies for Cu_6_Sn_5_ and Cu3Sn can be calculated by the Arrhenius relationship [40]:

*K = K*_0_ ∗ *exp(−Q/RT)*, (5)

where *K* is the growth constant (mm^2^/s), *K*_0_ is the frequency factor, *Q* is the activation energy, *R* is the gas constant (8.314 J/mol/*K*), and *T* is the aging temperature. The activation energies can be obtained from the slope of the ln(*K*) versus ln(*1/T*) plot. Figure 10 shows the Arrhenius plots of the interfacial IMC growth, and the activation energies values of Cu_6_Sn_5_ and Cu_3_Sn were 74.7 and 86.6 kJ/mol, respectively. This result is close to the values of 69.42 and 91.88 kJ/mol for Cu_6_Sn_5_ and Cu_3_Sn, respectively, in Sn3.0Ag0.5Cu/Cu solder joints during isothermal aging [41]. It was obvious that the activation energy of Cu_3_Sn was higher than that of Cu_6_Sn_5_, which indicated that the growth of Cu_3_Sn IMC was difficult.

The growth of interfacial Cu_3_Sn IMC depends on the temperature increase and the prolonged time. During the soldering process, the soldering time was so short that it could not provide the growth condition for Cu_3_Sn IMC with high activation energy. However, during the isothermal aging process, the heat preservation and long period satisfied the growth conditions of Cu_3_Sn. Therefore, the isothermal aging process provided growth conditions for Cu_3_Sn. This is in accordance with the formation of the Cu_3_Sn phase requiring an extended reaction time during the isothermal aging process [42]. It was reported that the growth rate of interfacial IMC is dependent on the activation energy when the supply of both the Sn and Cu atoms is sufficient [43]. During the soldering process, there was a liquid-state reaction, where the liquid phase Sn atoms of the soldering seam and solid-phase Cu atoms of the substrate were in full and direct contact with each other. The growth rate of interfacial Cu_6_Sn_5_ and Cu_3_Sn IMC mainly depends on the activation energy. However, during the isothermal aging process, the formation of Cu_6_Sn_5_ IMC after soldering may be a barrier for the diffusion of Cu and Sn atoms. In particular, the Sn atoms require long-range diffusion across the Cu_6_Sn_5_ IMC layer to react with the substrate Cu atoms to form a Cu_3_Sn IMC, which makes it more difficult for Cu_3_Sn IMC to grow. However, the growth rate of the Cu_3_Sn IMC was slightly faster than that of the Cu_6_Sn_5_ IMC during isothermal aging. This may be attributed to the Cu_3_Sn growth towards both the Cu substrate and solder seam sides. On the Cu_6_Sn_5_/Cu_3_Sn interface, the Cu_3_Sn grows at the expense of Cu_6_Sn_5_ according to reaction 1, while on the Cu_3_Sn/Cu interface, the growth of Cu_3_Sn can be expressed by reaction 2. The growth of Cu_6_Sn_5_ mainly depended on the reaction 6Cu + 5Sn = Cu_6_Sn_5_. The Cu atoms on the solder seam were in the form of a eutectic structure. A very small amount of free Cu atoms can diffuse to the Cu_6_Sn_5_/solder seam interface. At the same time, at the Cu_6_Sn_5_/Cu_3_Sn interface, the growth of Cu_3_Sn IMC consumed a certain amount of Cu atoms diffused from the Cu substrate, which may lead to a reduction in the amount of diffusion Cu atoms from the Cu substrate to the Cu_6_Sn_5_/solder seam interface, resulting in a limited growth rate of Cu_6_Sn_5_.

### 3.3. Mechanical Properties of the Sn2.5Ag0.7Cu0.1RE/Cu Solder Joints during Isothermal Aging

**Figure 11:**
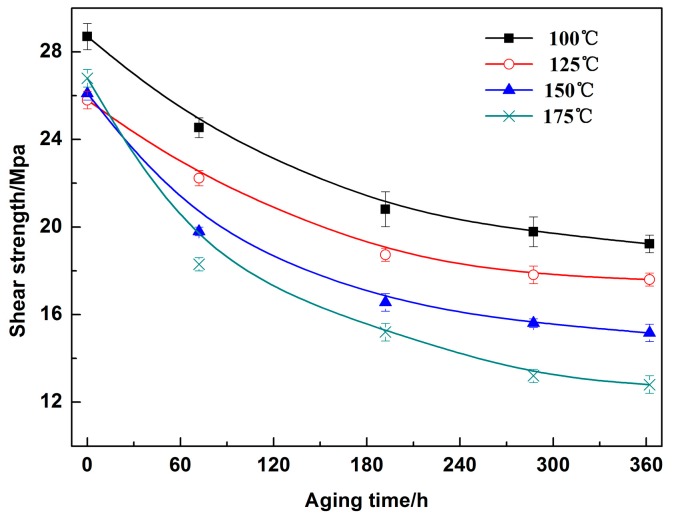
Shear strength of the solder joints for different aging time and temperature.

**Figure 12:**
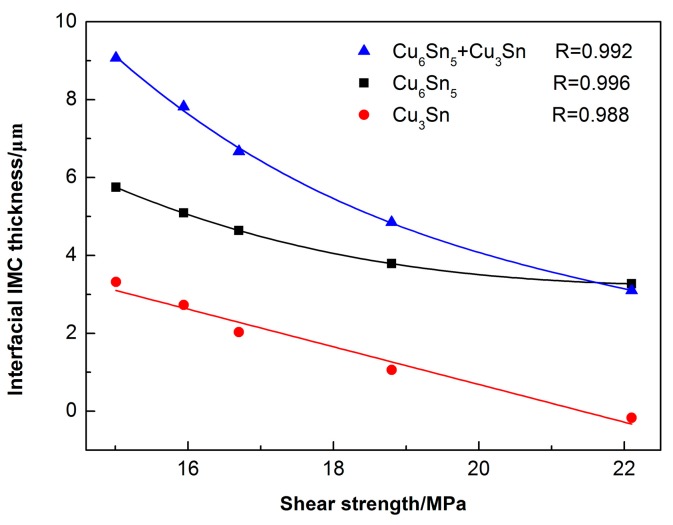
Relationship between shear strength and interfacial Cu_6_Sn_5_ and Cu_3_Sn thickness.

Figure 11 displays the shear strength of the Sn2.5Ag0.7Cu0.1RE/Cu solder joints for different isothermal aging times and temperatures. With the increase of isothermal aging time and temperature, the shear strength of the solder joints decreased, and the thickness of the interfacial IMC increased (Figure 7). Because of the brittleness of the Cu_3_Sn and Cu_6_Sn_5_ IMC, the increase in interfacial IMC thickness may deteriorate the mechanical properties of the solder joints. Additionally, during the isothermal aging process, the Kirkendall voids appeared in the Cu_3_Sn IMC, which is also harmful to the shear strength of the solder joint. To determine the relationship between the shear strength of the solder joints and interfacial IMC thickness, Figure 12 shows the multivariable regression analysis of the shear strength and thickness of the interfacial IMC. The shear strength of the solder joints was linearly correlated with the thickness of Cu_6_Sn_5_, which indicated that the thickness of Cu_3_Sn has a direct relation with the shear strength of the joints.

**Figure 13:**
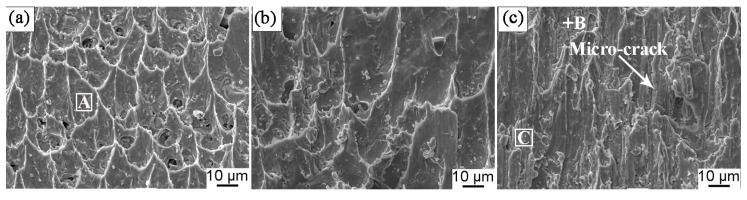
The shear fracture surface of the solder joints aged at 175 °C for a different time: (**a**) 0 h, (**b**) 192 h, and (**c**) 360 h.

**Figure 14:**
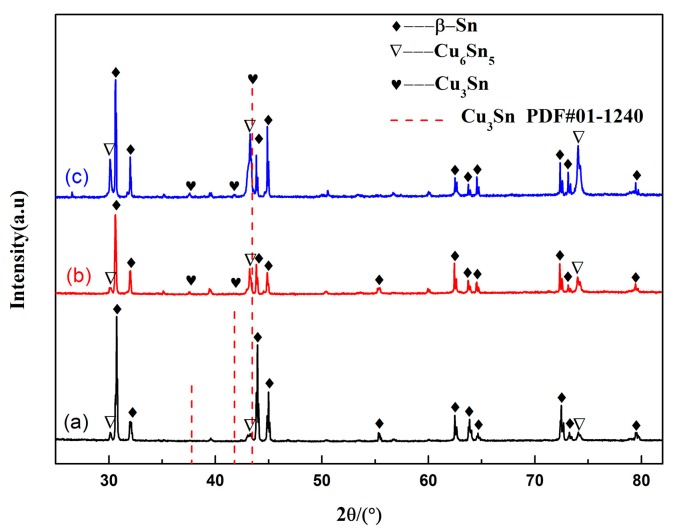
XRD analysis results of the fracture surface aged at 175 °C with different aging times: (**a**) 0 h, (**b**) 192 h, and (**c**) 360 h.

Figure 13 presents the shear fracture surface of the solder joints aged at 175 °C for a different time. The fracture surface displayed typical parabolic-shaped dimples after soldering, as shown in Figure 13a. These parabolic-shaped dimples indicated ductile fracture. With increasing aging time, the number of parabolic dimples decreased, and the cleavage planes appeared in the fracture surface after isothermal aging for 192 h as illustrated in Figure 13b. After aging for 360 h (Figure 13c), the fracture surface was dominated by the cleavage planes, and micro-cracks occurred, which indicated the brittle fracture manner. The composition of “A”, “B”, and “C” areas are listed in Table 1. The area “A” contained mainly Sn, whereas the areas “B” and “C” consisted of Sn and Cu, and the mole ratio of Cu to Sn was approximately 3:1 and 6:5, respectively. Thus, the cleavage plane may be the Cu_6_Sn_5_ and Cu_3_Sn IMC. We can deduce that with increasing isothermal aging time, the fracture mechanism transformed from the ductile-type fracture mechanism with the dominant parabolic-shaped dimples in the solder seam to brittle fracture with the cleavage of the interfacial Cu_6_Sn_5_ and Cu_3_Sn IMC. Figure 14 shows the XRD pattern of the fracture surface of the solder joints aged at various times. Compared with the sample after soldering, the intensity of the Cu_6_Sn_5_ peak is higher, and the ratio of the Cu_6_Sn_5_ to β-Sn peak becomes gradually higher with increasing isothermal aging time. Additionally, the intensity of the Cu_3_Sn peak increased with prolonged aging time. This indicated that the fracture pathway transferred from the solder seam to the interfacial IMC, which was near the side of the Cu_3_Sn IMC layer. This result is consistent with the above shear fracture morphology analysis. The change in the fracture pathway was mainly attributed to the increase of interfacial IMC thickness during the isothermal aging process. In general, the coefficient of thermal expansion (CTE) between the interfacial IMC layer (1.84 × 10^−5^ m/K) and Cu substrate (1.67 × 10^−6^ m/K) was mismatched, leading to a stress concentration during long isothermal aging [44]. Hence, the micro-cracks occurred and propagated along with the interface. In addition, we also found that with increasing isothermal aging temperature, the fracture pathway shifted from the solder seam to the direction of the interfacial IMC layer.

## 4. Conclusions

In this study, the microstructure evolution of the interfacial IMC and the mechanical properties of Sn2.5Ag0.7Cu0.1RE/Cu solder joints during the isothermal aging process were investigated by dynamic observation. The conclusions can be summarized as follows.

1. During the isothermal aging process of Sn2.5Ag0.7Cu0.1RE/Cu lead-free solder joints, interfacial IMC evolved from single Cu_6_Sn_5_ to Cu_6_Sn_5_ and Cu_3_Sn double layers. The Cu_3_Sn grew towards the Cu substrate and the solder seam sides; growth toward the Cu substrate side was dominant. The growth of the Cu_3_Sn IMC was depended on prolonged time at a certain temperature. Additionally, the growth of the interfacial IMC was mainly controlled by the bulk diffusion mechanism, and the activation energy values of Cu_6_Sn_5_ and Cu_3_Sn were 74.7 and 86.6 kJ/mol, respectively. The growth rate of Cu_3_Sn was slightly faster than that of Cu_6_Sn_5_ during the isothermal aging process.

2. With increasing isothermal aging time and temperature, the shear strength of the solder joints decreased, which was linearly related to the thickness of the interfacial Cu_3_Sn IMC. Additionally, the fracture mechanism of the solder joints changed from ductile fracture, which contained dimples, to brittle fracture, which contained cleavage planes; the fracture pathway also moved from the solder seam to the interfacial IMC layer, which was close to the Cu_3_Sn IMC.

## Figures and Tables

**Table 1:** EDS analysis results of the “A”, “B”, and “C” areas in Figure 13.

Area	Mole Fraction/%		
	Sn	Cu	Ag
A	93.49	5.34	1.18
B	24.72	75.28	-
C	46.64	53.36	-
